# Antimicrobial potential of silver nanoparticles synthesized from mushroom extracts against drug-resistant pathogens

**DOI:** 10.1016/j.nmni.2026.101716

**Published:** 2026-01-28

**Authors:** Mehrajud Din Talie, Nusrat Ahmad, Mansoor Ahmad Malik, Abdul Hamid Wani, Mohd Yaqub Bhat

**Affiliations:** Section of Mycology and Plant Pathology, Department of Botany, University of Kashmir, Srinagar, 190006, J&K, India

**Keywords:** Mycosynthesis, Ascomycetous mushrooms, Nanofungicides, Nanobactericides

## Abstract

Green synthesis of silver nanoparticles (AgNPs) has emerged as a sustainable alternative to conventional chemical methods due to the presence of natural bioactive compounds in biological systems. Although Ascomycetous macrofungi are rich sources of antibacterial and antioxidant metabolites, their potential in nanoparticle synthesis remains largely unexplored. In the present study, silver nanoparticles were synthesized for the first time using extracts of the Ascomycetous mushrooms *Geopora sumneriana* and *Verpa bohemica* as eco-friendly reducing and capping agents. The biosynthesized AgNPs were characterized using UV–visible spectroscopy, field emission scanning electron microscopy (FESEM), X-ray diffraction (XRD), and Fourier transform infrared (FTIR) spectroscopy. UV–visible analysis confirmed nanoparticle formation through a characteristic surface plasmon resonance band at ∼420–450 nm, while FESEM revealed predominantly spherical to hexagonal nanoparticles. XRD analysis confirmed the crystalline nature of AgNPs with a face-centered cubic structure, and FTIR results indicated the involvement of fungal biomolecules such as proteins, phenolics, and polysaccharides in nanoparticle reduction and stabilization. The biosynthesized AgNPs were subsequently evaluated for their antimycotic and antibacterial efficacy, including minimum inhibitory concentration (MIC) determination, against a range of phytopathogenic fungi and clinically relevant bacterial strains. The AgNPs exhibited strong antifungal and antibacterial activities against *Aspergillus niger*, *Penicillium chrysogenum*, *Pythium ultimum*, *Escherichia coli*, *Salmonella gallinarum*, and *Staphylococcus aureus*. The MIC values of AgNPs synthesized using *Verpa bohemica* ranged from 0.9 to 1.0 mg/mL for fungal strains and 0.7–0.8 mg/mL for bacterial strains. Similarly, AgNPs derived from *Geopora sumneriana* showed MIC values ranging from 0.6 to 1.1 mg/mL for fungi and 0.4–0.8 mg/mL for bacteria. The pronounced growth inhibition of diverse pathogenic microorganisms underscores the strong antimicrobial potential of these mycosynthesized AgNPs. Overall, the study highlights *Geopora sumneriana* and *Verpa bohemica* as efficient biogenic nanofactories and demonstrates the potential of mycosynthesized AgNPs as eco-friendly nanofungicides and nanobactericides for controlling multidrug-resistant pathogens in agriculture, aquaculture, and horticulture.

## 1 Introduction

Nanoscience, a brainchild of modern materials science, is a rapidly growing and multidisciplinary field that unites the efforts of physicists, chemists, biologists, physicians, materials scientists, computer scientists, mathematicians, and others [[1], [2], [3]]. Over the last century, there has been a marked increase in the application of metal nanoparticles across numerous fields, including biomedicine, food, agriculture, horticulture, aquaculture, biotechnology, consumer product industries, engineering, electronics, energy, optics, drug delivery systems, catalysis, and cosmetics [[4], [5], [6], [7]]. To date, nanoparticles have traditionally been synthesized using physical and chemical approaches [[8], [9], [10]]. However, physical methods are costly, yield low production, and require sophisticated instrumentation, whereas chemical methods involve toxic substances as reducing and stabilizing agents, posing serious environmental and health hazards [11,12].

In recent years, green synthesis approaches for nanoparticle production, particularly silver nanoparticles (AgNPs), have been developed worldwide [[13], [14], [15], [16], [17]]. Biological approaches outperform conventional methods due to their lower toxicity, abundant availability of raw materials, cost-effectiveness, procedural simplicity, biocompatibility, rapid synthesis, improved control over morphology, eco-friendliness, and higher safety and efficiency [[18], [19], [20]]. In these biological methods, extracts of fungi, bacteria, algae, and plants are used as reducing and stabilizing agents for the synthesis of silver and other nanoparticles [[21], [22], [23], [24], [25], [26], [27]]. Furthermore, nanoparticles synthesized through green approaches are reported to be more biocompatible and biologically active than those produced via chemical or physical methods [28,29].

Microbial infections pose a serious threat to agriculture, horticulture, aquaculture, and healthcare sectors worldwide. Moreover, the increasing prevalence of multidrug-resistant microorganisms has intensified the need for novel antibacterial and antifungal agents with multipurpose characteristics such as low toxicity, high biocompatibility, and strong antimicrobial efficacy. In this context, biosynthesized silver nanoparticles have gained global attention due to their high surface-to-volume ratio and strong electrostatic interactions with microbial cells, which result in enhanced antimicrobial properties [[30], [31], [32]]. Recently, several studies have reported the antimycotic and antibacterial activities of nanoparticles [[33], [34], [35], [36], [37]]. The antimicrobial efficacy of AgNPs is attributed to multiple synergistic mechanisms, including strong electrostatic interactions between positively charged AgNPs and negatively charged microbial cell membranes, leading to membrane destabilization and increased permeability [34,35]. Furthermore, AgNPs and released Ag^+^ ions penetrate microbial cells and interact with intracellular proteins, thiol-containing enzymes, and nucleic acids, resulting in protein denaturation, inhibition of DNA replication, and disruption of essential metabolic pathways [[38], [39], [40]]. AgNPs are also known to induce the generation of reactive oxygen species (ROS), which cause oxidative stress, lipid peroxidation, and irreversible damage to cellular components, ultimately leading to cell death [[41], [42], [43]]. The multi-target mode of action of AgNPs significantly reduces the likelihood of resistance development, making them promising alternatives to conventional antibiotics [44,45].

Among biological systems, fungi have attracted considerable attention due to their ability to secrete large amounts of proteins compared to bacteria, which play a crucial role in nanoparticle reduction and stabilization [38]. Several microfungi, including *Rhizopus arrhizus*, *Fusarium oxysporum*, *Aspergillus terreus*, *Penicillium* spp., *A. niger*, *Neurospora crassa*, and *Trichoderma gamsii*, have been explored for the green synthesis of silver nanoparticles [24,39,40]. Similarly, numerous macrofungi, primarily belonging to Basidiomycetes—such as *Pleurotus* spp., *Agaricus* spp., *Ganoderma* spp., *Fomes fomentarius*, and *Flammulina velutipes*—have also been reported for AgNP biosynthesis [[41], [42], [43], [44], [45]].

Likewise, Ascomycetous mushrooms are among the most extensively collected and consumed higher fungi due to their high nutritional and medicinal value, and they are rich sources of antioxidants, anticancer, antimicrobial, and other bioactive compounds. Despite these attributes, only a limited number of studies have explored the biosynthesis of silver nanoparticles using Ascomycetous macrofungi [20]. Therefore, it is imperative to identify novel macrofungal species capable of fabricating AgNPs and to investigate their potential applications in plant and human disease management strategies. Despite extensive research on green synthesis of silver nanoparticles using plants, bacteria, and basidiomycetous fungi, the exploitation of Ascomycetous macrofungi for nanoparticle fabrication remains largely unexplored. In particular, there are no detailed reports on the mycosynthesis of silver nanoparticles using *Geopora sumneriana* and *Verpa bohemica*, nor on the mechanistic basis of their antimicrobial activity. The present study addresses this critical knowledge gap by reporting, for the first time, the eco-friendly synthesis of silver nanoparticles mediated by extracts of these underutilized Ascomycetous mushrooms. In addition to comprehensive physicochemical characterization, this work provides mechanistic insights into the antibacterial and antimycotic action of the biosynthesized nanoparticles. By integrating fungal biodiversity with sustainable nanotechnology, the study establishes a novel and efficient biofabrication platform with potential applications in plant and human disease management.

## 2 Materials and methods

### 2.1 Collection of ascocarps

The Ascomycetous mushrooms used as reducing agents were collected from coniferous forests of Northern Kashmir. They were recognized and identified, based on micro-morphological and molecular description using analysis of ITS sequences [46,47]. The ascocarps were cleaned, air-dried and then ground to fine powder. Besides, the silver nitrate (≥99.0 %, AgNO3) was purchased from Supreme Syndicate, Srinagar.

### 2.2 Preparation of mushroom extracts

In 200 ml of distilled water, 15 g of the blended sample was suspended and boiled for 05–10 min. The extract was allowed to cool to room temperature before being shaken for 24 h on an orbital shaker. The mixture was filtered two to three times and stored at 4 °C for further use.

### 2.3 Synthesis of silver nanoparticles

For mycosynthesis, the methods of Narasimha et al. [48] and Talie et al. [20] were followed with little modifications. In brief, 50 ml of mushroom extracts were mixed with 50 ml of (0.1 mM) silver nitrate solution, followed by incubation at room temperature for the bio-reduction process. The conversion of the colour less solution to a brown coloured solution indicates that the bio-reduction was completed, from Ag ^+^ to Ag^o^.

### 2.4 Physicochemical characterization

#### 2.4.1 UV–visible spectroscopic studies

The optical absorption analysis of the bio-reduced mixture was scrutinized using UV–Visible (UV-119 Systronics) spectrophotometer at room temperature in between wavelength of 300–700 nm, and spectral data were plotted on Origin (version 2021b) software.

#### 2.4.2 Field Emission scanning Electron Microscopic (FESEM) studies

The morphology of mycosynthesized AgNPs were assessed by SEM analysis. The AgNPs were biosynthesized using Ascomycetous mushroom extracts and pulverized to a fine powder after being exposed to complete aridity. The powdered nanomaterials were then gold coated and analysed using a FESEM (GeminiSEM 500) instrument at IIIM Jammu and NIT, Srinagar and images were recorded.

#### 2.4.3 X-Ray Diffraction (XRD) analysis

The diffraction outline was documented to study the structure as well as composition in the range of 20 to 90θ. The high-quality pulverized AgNPs were mounted on XRD slide and characterized under XRD (SmartLab Rigaku) instrument at NIT, Srinagar. Besides, the powdered material of AgNPs was evaluated by XRD analysis to confirm their crystallinity.

#### 2.4.4 Fourier Transmission Infrared (FTIR) spectroscopy

FTIR investigation was achieved to identify the functional groups of numerous biomolecules present in the Ascomycetous mushroom extract, which were involved in reducing AgNO_3_and stabilization of AgNPs. The finely pulverized AgNPs were subjected to FTIR analysis with a broker (Alpha 200486) instrument in the frequency range of 4000 cm^−1^ to 500 cm^−1^.

### 2.5 Antimicrobial assay

The antifungal and antibacterial activities of mycosynthesized silver nanoparticles using Ascomycetous mushrooms were assessed against various pathogens by establishing diverse concentrations.

#### 2.5.1 Test organisms

At the Section of Mycology and Plant Pathology, Department of Botany, University of Kashmir, Srinagar (190006), J&K, India, the pathogenic fungi utilised in this work were successfully identified from spoiled or rotted apple samples. The harmful bacterial strains that cause various infections, on the other hand, were obtained online from Chandigarh, India's Institute of Microbial Technology (IMTECH). The names and sources of the pathogens that were examined are included in Table 1.

**Table 1:** List of fungal and bacterial strains screened for antimicrobial properties, along with their names and sources.

S. No.	Test organism	Source
01	*Penicillium chrysogenum*	Section of Mycology and Plant Pathology Lab., UOK, Srinagar, India.
02	*Aspergillus niger*	Section of Mycology and Plant Pathology Lab., UOK, Srinagar, India.
03	*Pythium ultimum*	Section of Mycology and Plant Pathology Lab., UOK, Srinagar, India.
04	*Staphylococcus aureus*	Institute of Microbial Technology (MTCC-96), Chandigarh, India.
05	*Salmonella gallinarum*	Institute of Microbial Technology (MTCC- 1162), Chandigarh, India.
06	*Escherichia coli*	Institute of Microbial Technology (MTCC-407), Chandigarh, India.

#### 2.5.2 Agar well diffusion method

The antibacterial and antifungal activities of mycosynthesized silver nanoparticles and aqueous extracts were determined using agar well diffusion approach of Wiegand et al. [49].

#### 2.5.3 Antibacterial sensitivity assay

The bacterial strains purchased were sub-cultured on fresh nutrient broth medium and were then placed on orbital shaker. The Muller Hinton Agar (MHA) medium was prepared, autoclaved, poured on sterilized petri plates and allowed to solidify under laminar hood. The petri dishes were then inoculated by the freshly prepared microbial suspension with the help of inoculation loop and wells were prepared by 5 mm cork borer. The wells were filled with different concentrations of biosynthesized silver nanoparticles, viz., 10 mg/ml, 15 mg/ml and 20 mg/ml. As a positive control or a standard, gentamycin discs (50mcg/disc) were utilised. After that, the cultures were wrapped and incubated for 24 h at 37±2^o^C.The zones of inhibition in millimetres were measured following incubation, and each experiment was achieved in triplicates.

#### 2.5.4 Antifungal assay

Antimycotic activity was assessed using one-week-old fungal cultures maintained on PDA medium. In culture tubes, a 0.02 ml inoculum of each fungal pathogen was injected in 20 ml of molten Sabouraud dextrose agar (SDA) medium. After homogenising the culture tubes, they were placed onto 90 mm petri plates. Upon allowing the cultures to solidify in the laminar airflow, wells were drilled using a 5 mm standard cork borer. The wells were filled with biosynthesized silver nanoparticles at concentrations of 10 mg/ml, 15 mg/ml, and 20 mg/ml. As a positive control, Nystatin (50 mcg/disc) discs were employed. After that, the plates were wrapped and maintained for two days at 25±2^o^C.By calculating the diameter of inhibition zones in millimetres, the antimycotic activity of mycosynthesized silver nanoparticles was calculated.

#### 2.5.5 Determination of minimum inhibitory concentrations (MIC)

In the present study, MIC values were assessed for the biosynthesized silver nanoparticles which exhibited significant antibacterial and antifungal properties when evaluated by agar well diffusion assay. The MIC values were determined by the methods of Wiegand et al. [49] and Iqbal et al. [50] with little modifications.

### 2.6 Statistical analysis

The experimentations were accomplished in triplicates. Data was statistically analysed using SPSS 16.0 software; mean and standard deviation were calculated. ANOVA was used to test the differences between different treatments. Duncan's multiple comparison tests were used to compare all the treatments and differences between individual means at *P* ≤ 0.05. The results were expressed as mean values and standard deviation. Besides, Origin (version 2021b) software was used to plot spectral graphs.

## 3 Results

Macrofungal extracts serve as a beneficial platform for enlightening the green synthesis approach of silver nanoparticle synthesis. But, the alterations in biochemical composition of different mushrooms may expressively disturb the mycosynthesis procedure [11,51]. In the present study, AgNPs were effectively synthesized employing a green approach using Ascomycetous mushrooms, and their antifungal and antibacterial properties were characterised and analysed. The following sections go through the findings of our research in further depth.

### 3.1 Visual and UV–visible study

**Fig. 1:**
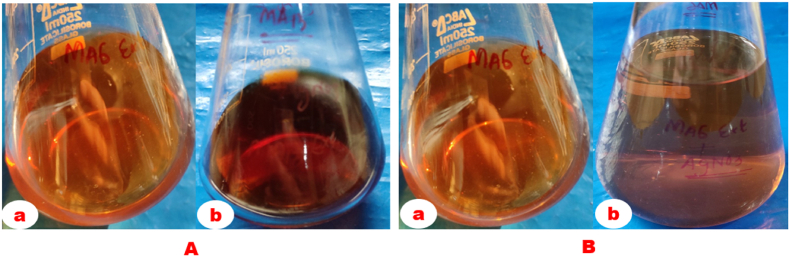
(A) *Verpa bohemica*; B) *Geopora sumneriana*: (a) Before addition of silver nitrate solution mushroom extract (b) After addition of silver nitrate to mushroom extract.

**Fig. 2:**
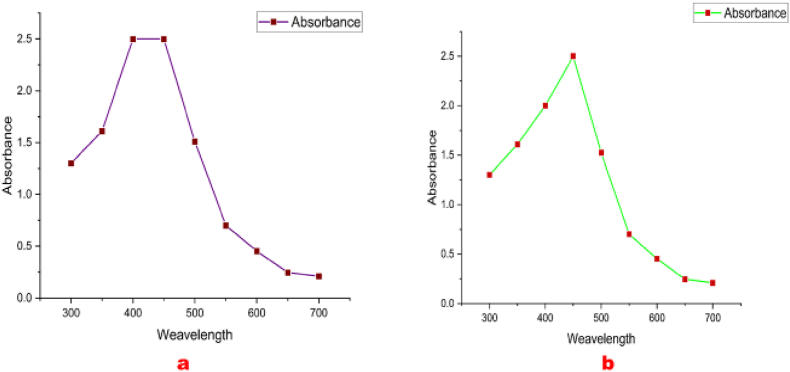
UV–Visible spectroscopy of mycosynthesized silver nanoparticles (AgNPs) using Ascomycetous mushroom extracts (a) *Verpa bohemica* (b) *Geopora sumneriana.*

Visual observations of a transition in color from pale yellow to dark brown within 24 h of incubation validated the primary signs of mycosynthesis of AgNPs utilising Ascomycetous mushroom extracts (Fig. 1). Likewise, the bio-reduction of aqueous Ag^+^ ions by Ascomycetous mushroom extracts were observed by spectroscopic investigation in between the frequencies of 300–700 nm. Substantial plasmonic absorption bands were identified at 420–450 nm (Fig. 2), demonstrating that *Verpa bohemica* and *Geopora sumneriana* mushroom extracts were used to successfully mycosynthesized AgNPs.

### 3.2 SEM analysis

**Fig. 3:**
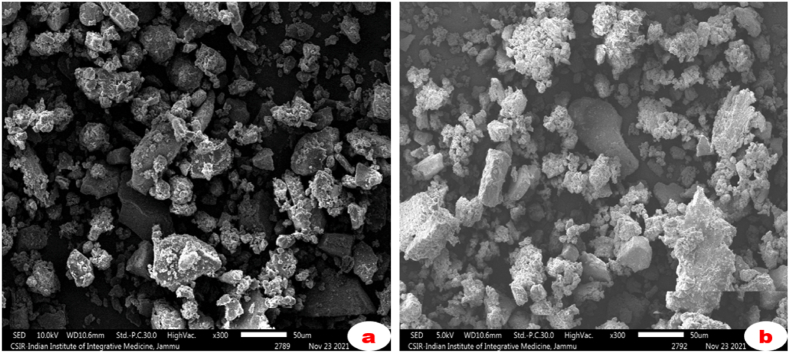
Silver nanoparticles mycosynthesized using Ascomycetous mushroom extracts, as seen in SEM micrographs (a) *Verpa bohemica* (b) *Geopora sumneriana.*

The surface morphology and size of the mycosynthesized silver nanoparticles were studied using SEM studies. The findings (Fig. 3) demonstrated that AgNPs biosynthesized using *Verpa bohemica* and *Geopora sumneriana* mushroom extracts were approximately spherical or hexagonal in shape and mostly in aggregated form. Besides, the particle size ranged in between 30 and 50 nm.

### 3.3 XRD study

**Fig. 4:**
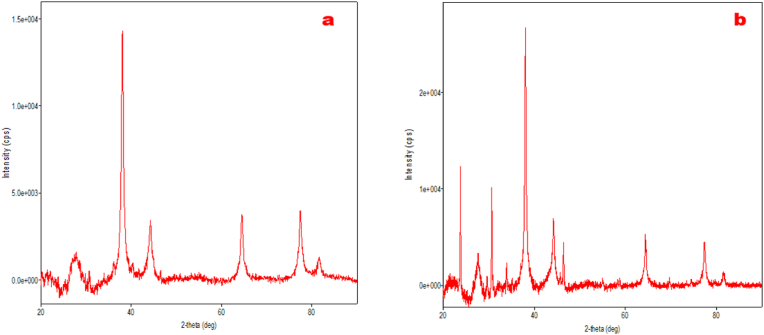
X-ray diffraction (XRD) spectrum of mycosynthesized AgNPs using Ascomycetous mushrooms (a) *Verpa bohemica* (b) *Geopora sumneriana.*

The crystalline nature of mycosynthesized silver nanoparticles using Ascomycetous mushrooms were verified by XRD measurements (Fig. 4) which exhibited peaks at 38.04^o^, 44.06^o^,64.34^o^ and 77.17^o^ that are analogous to the intensities of 111, 200, 220 and311 reflection planes, respectively.

### 3.4 FTIR study

**Fig. 5:**
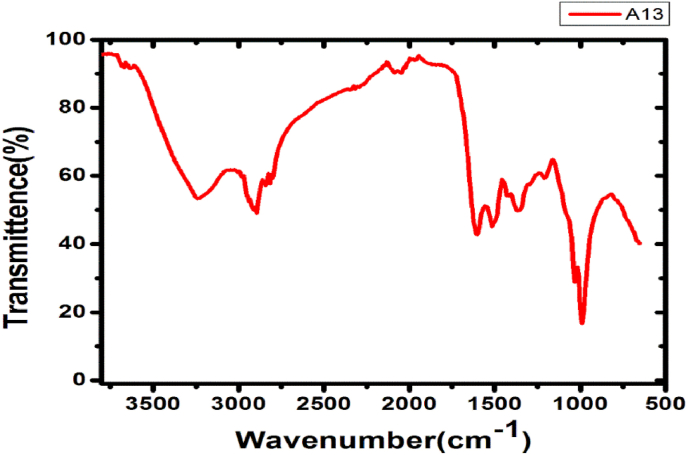
Fourier transmission infrared spectra of AgNPs mycosynthesized employing *Verpa bohemica* mushroom extract.

To study the core-shell morphology and crucial information regarding potential biomolecules responsible for mycosynthesis of silver nanoparticles, FTIR serve as a valuable tool. FTIR examination of nanoparticles prepared using *Verpa bohemica* mushroom extract exhibited excellent absorption bands at 1050.00, 1105.42, 1384.41, 1522.06, 1600–2000, 2851.98, 2937.59, 3300.00, and 3452.06 cm^−1^ (Fig. 5). The C-O stretch of carbs or alcohol is linked to the 1050.00 cm^−1^ absorption band, the C-O stretch of proteins is linked to the 1105.42 cm^−1^ absorption band, the O-H of phenol or the C-N stretch of aromatic amines is linked to the 1384.41 cm^−1^ absorption band, and the C=N or C=C of primary or secondary amines is linked to the 1522.06 cm^−1^absorption band. Accordingly, aromatic combination bands are linked to several absorption bands in the 1600-2000 cm^−1^ range, and the 2851.98 cm^−1^ absorption band correlates to CH_2_ stretching of lipids or fats.

Besides, the N-H imino stretching was revealed by the 3300.00 cm^−1^ absorption band. As a result, phenols, proteins, and amines are implicated in the stabilization of mycosynthesized AgNPs prepared from *Verpa bohemica* mushroom extracts in the current study.

**Fig. 6:**
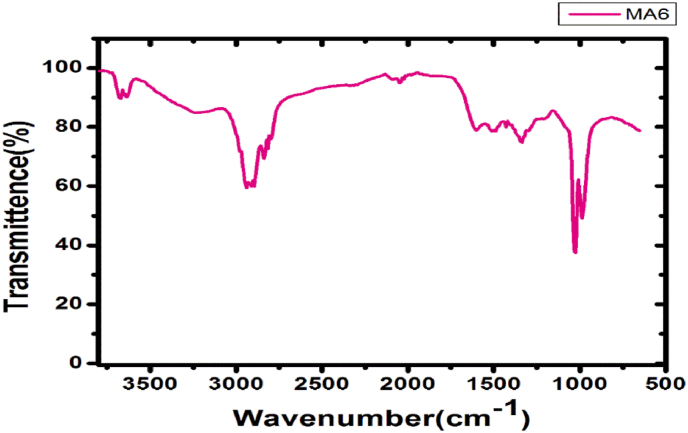
Fourier transmission infrared spectra of AgNPs mycosynthesized employing *Geopora sumneriana* mushroom extract.

In addition, biosynthesized nanoparticles prepared employing *Geopora sumneriana* mushroom extract showed potential absorption bands in the whole FTIR spectrum extending from 500 to 3600 cm^−1^ (Fig. 6). The C-O stretch of carbs has an absorbance peak of 1053.06 cm^−1^, while the PO_2-_of proteins or the CN stretch of tertiary amines has an absorption band of 1230.00 cm^−1^.Similarly, the NH component of amines is correlated to the absorption bands of 2332.00 and 2370.87 cm^−1^, whereas the CH_2_ and CH_3_ stretches of lipids or fats are linked to the absorption bands of 2857.96 and 2937.59 cm^−1^. As a result of the current study, carbs, proteins, and amines are assumed to be involved in the stabilization of AgNPs mycosynthesized by *Geopora sumneriana* mushroom extract.

### 3.5 Antibacterial activity

**Fig. 7:**
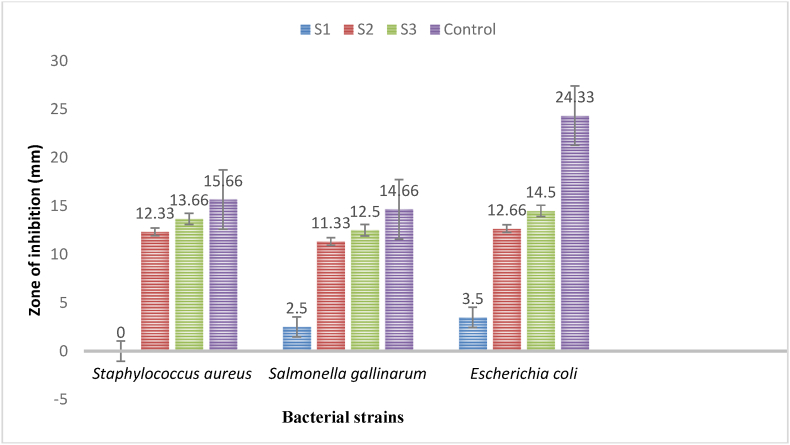
Antibacterial activities of biosynthesized AgNPs using *Geopora sumneriana* against pathogenic bacterial strains.

**Fig. 8:**
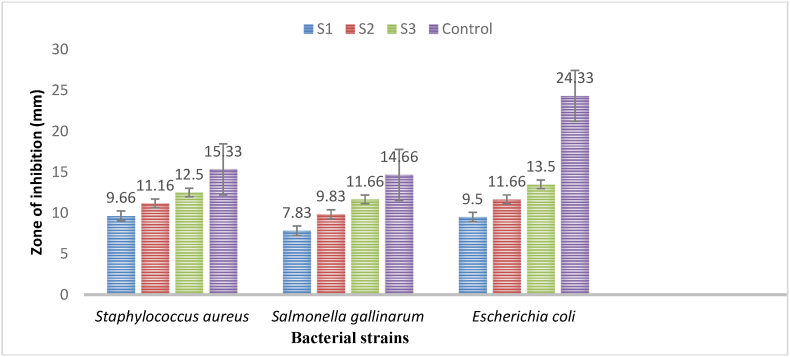
Antibacterial effects of mycosynthesized silver nanoparticles using *Verpa bohemica* mushroom extracts on tested bacterial strains.

**Fig. 9:**
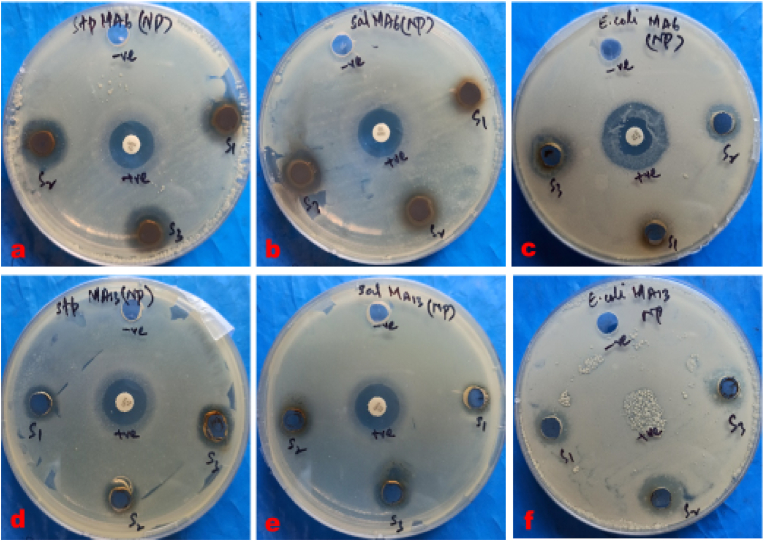
Antibacterial activities of silver nanoparticles synthesized from *Geopora sumneriana* against a) *Staphylococcus aureus,* (b) *Salmonella gallinarum*, (c) *Escherichia coli;* Antibacterial activities of silver nanoparticles synthesized from *Verpa bohemica* against: (d) *Staphylococcus, e) Salmonella gallinarum,* (f) *Escherichia coli*.

The antibacterial assay results (Table 2, Fig. 7, Fig. 9) revealed that highest concentrations of synthesized AgNPs using *Geopora sumneriana* exhibited maximum zone of inhibition against *Escherichia coli* (14.50 ± 0.86 mm) followed by *Staphylococcus aureus* (13.66 ± 0.57 mm) and *Salmonella gallinarum* (12.50 ± 0.50 mm), respectively. However, lowest concentrations also revealed inhibitory effects against all the tested pathogenic bacterial strains but to a minor degree. Likewise, the findings (Table 2, Fig. 8, Fig. 9) verified that the highest concentrations of synthesized AgNPs using *Verpa bohemica* exhibited maximum zone of inhibition against *Escherichia coli* (13.50 ± 0.50 mm) followed by *Staphylococcus aureus* (12.50 ± 0.50 mm). However, minimum antibacterial activity was reported against *Salmonella gallinarum* (11.66 ± 0.57 mm) at higher concentrations.

**Table 2:** Effect of biosynthesized AgNPs using *Geopora sumneriana* and *Verpa bohemica* on zone of inhibition against different pathogenic bacteria.

S.No	Bacterial species	Zone of inhibition (mm)			
		10 mg/ml	15 mg/ml	20 mg/ml	Gentamycin
***Geopora sumneriana***	*Staphylococcus aureus*	10.83 ± 0.76^d^^,^^a^	12.33 ± 0.57^c^	13.66 ± 0.57^b^	15.66 ± 0.57^a^
	*Salmonella gallinarum*	09.66 ± 0.57^d^	11.33 ± 0.57^c^	12.50 ± 0.50^b^	14.66 ± 0.57^a^
	*Escherichia coli*	10.66 ± 0.57^d^	12.66 ± 0.57^c^	14.50 ± 0.86^b^	24.33 ± 0.57^a^
***Verpa bohemica***	*Staphylococcus aureus*	09.66 ± 0.57^d^	11.16 ± 0.28^c^	12.50 ± 0.50^b^	15.33 ± 0.57^a^
	*Salmonella gallinarum*	07.83 ± 0.76^d^	09.83 ± 0.76^c^	11.66 ± 0.57^b^	14.66 ± 0.57^a^
	*Escherichia coli*	09.50 ± 0.50^d^	11.66 ± 0.57^c^	13.50 ± 0.50^b^	24.33 ± 0.57^a^

a Values are represented as mean ± SD of three replicates. The Duncan multiple comparison test was used to compare mean values.

### 3.6 Antifungal activity

**Fig. 10:**
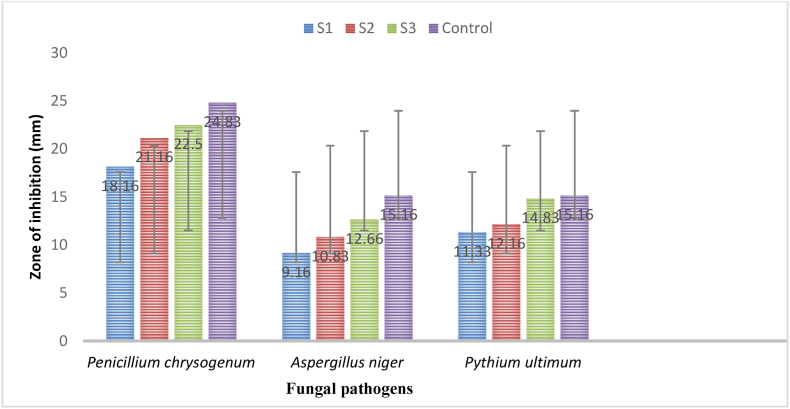
Antimycotic activity of mycosynthesized silver nanoparticles using *Geopora sumneriana* mushroom extraction different tested fungal pathogens.

**Fig. 11:**
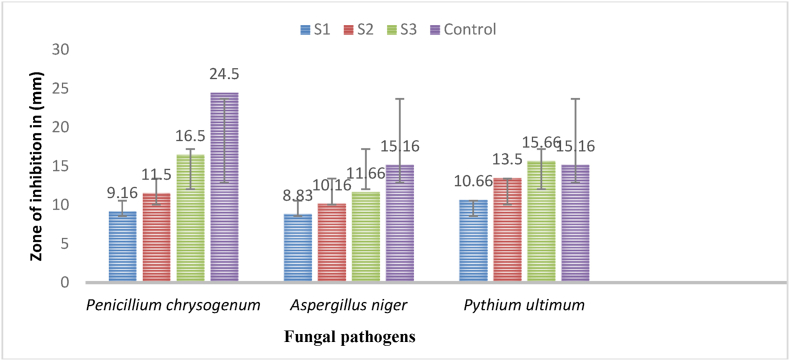
Antifungal activity of biosynthesized silver nanoparticles using *Verpa bohemica* against tested fungal pathogens.

**Fig. 12:**
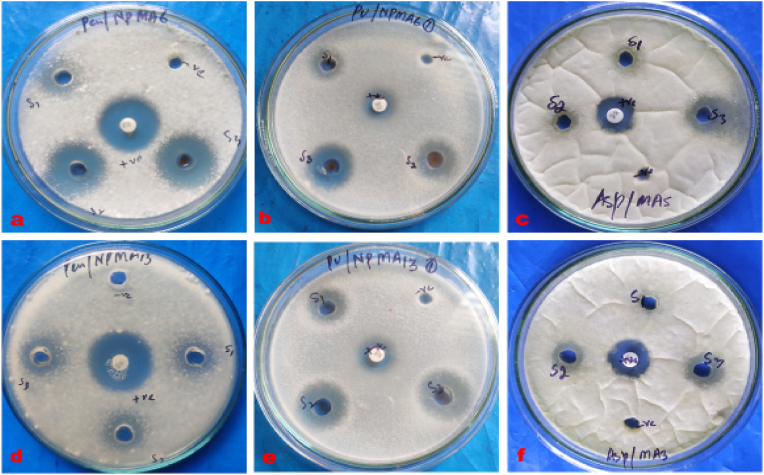
Antifungal activities of silver nanoparticles synthesized from *Geopora sumneriana* against: (a) *Penicillium chrysogenum*, (b) *Pythium ultimum,* (c) *Aspergillus niger;* Antifungal activities of silver nanoparticles synthesized from *Verpa bohemica* against: *(*d) *Penicillium chrysogenum*, (e) *Pythium ultimum,* (f) *Aspergillus niger*.

In the study, the findings (Table 3, Fig. 10, Fig. 12) confirmed that the highest concentrations of biosynthesized AgNPs using *Geopora sumneriana* mushroom extract were more effective than lower concentrations in causing zone of inhibition. Furthermore, highest concentrations presented maximum inhibition zone on *Penicillium chrysogenum* (22.50 ± 0.50 mm) followed by *Pythium ultimum* (14.83 ± 0.28 mm) and *Aspergillus niger* (12.66 ± 0.28 mm), respectively. Similarly, the antimycotic results revealed (Table 3, Fig. 11, Fig. 12) that all the dosses of AgNPs biosynthesized using *Verpa bohemica* showed substantial inhibitory effects against all the tested fungi. However, uppermost concentrations exhibited maximum inhibition zones against *Penicillium chrysogenum* (24.00 ± 1.00 mm) followed by *Aspergillus* (20.33 ± 0.57 mm) and *Pythium ultimum* (19.33 ± 1.54 mm), respectively. Besides, it was observed from the results that highest dosses of mycosynthesized AgNPs resulted into potent inhibitory effects against *Pythium ultimum* as compared to the positive control utilised in the present study. Therefore, the biosynthesized AgNPs act as a potent antifungal agent against *Pythium ultimum* than other tested fungi.

**Table 3:** Effect of mycosynthesized AgNPs using *Geopora sumneriana* and *Verpa bohemica* on mycelial progress of different pathogenic fungi.

S.No	Fungal species	Zone of inhibition (mm)			
		10 mg/ml	15 mg/ml	20 mg/ml	Nystatin
***Geopora sumneriana***	*Penicillium chrysogenum*	18.16 ± 0.76^d^^,^^a^	21.16 ± 0.76^c^	22.50 ± 0.50^b^	24.83 ± 0.28^a^
	*Aspergillus niger*	09.16 ± 0.28^d^	10.83 ± 0.28^c^	12.66 ± 0.28^b^	15.16 ± 0.28^a^
	*Pythium ultimum*	11.33 ± 0.28^d^	12.16 ± 0.28^c^	14.83 ± 0.28^b^	15.16 ± 0.28^a^
***Verpa bohemica***	*Penicillium chrysogenum*	09.16 ± 0.28^d^	11.50 ± 0.50^c^	16.50 ± 0.50^b^	24.50 ± 0.50^a^
	*Aspergillus niger*	08.83 ± 0.28^d^	10.16 ± 0.28^c^	11.66 ± 0.28^b^	15.16 ± 0.28^a^
	*Pythium ultimum*	10.66 ± 0.57^d^	13.50 ± 0.50^c^	15.66 ± 0.28^b^	15.16 ± 0.28^a^

a Values are represented as mean ± SD of three replicates. The Duncan multiple comparison test was used to compare mean values.

### 3.7 Minimum inhibitory concentration

**Fig. 13:**
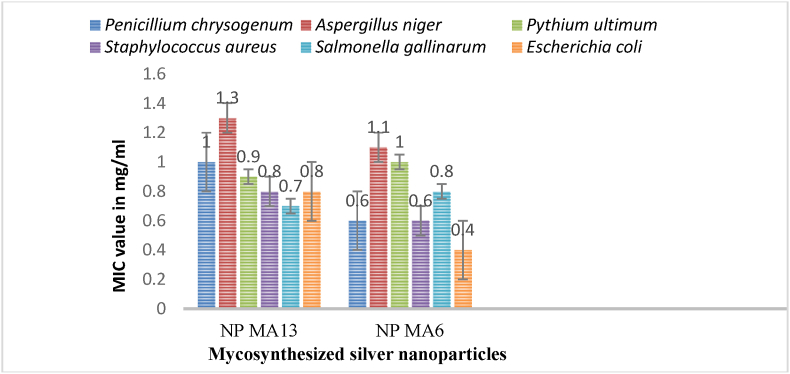
Minimum inhibitory concentration (MIC) of mycosynthesized silver nanoparticles using *Verpa bohemica* (NP MA13) and *Geopora sumneriana* (NP MA6) mushroom extracts for the tested fungal and bacterial pathogenic strains.

The findings (Table 4 and Fig. 13) demonstrated that the AgNPs synthesized from *Verpa bohemica* mushroom revealed the MIC values of 1.0, 1.3, and 0.9 mg/ml against tested fungal strains such as *Penicillium chrysogenum, Aspergillus niger* and *Pythium ultimum* while as the said nanomaterials showed 0.8, 0.7 and 0.8 mg/ml MIC values for some tested bacterial strains, namely *Staphylococcus aureus, Salmonella gallinarum* and *Escherichia coli*. Similarly, it was quite evident from the results that synthesized AgNPs from *Geopora sumneriana* mushroom extract exhibited MIC value of 0.6 mg/ml for *Penicillium chrysogenum,* 1.1 mg/ml for *Aspergillus niger* and 1.0 mg/ml for *Pythium ultimum* while as MIC values of 0.6, 0.8 and 0.4 mg/ml were reported against *Staphylococcus aureus, Salmonella gallinarum* and *Escherichia coli*, respectively.

**Table 4:** Minimum inhibitory concentration of synthesized silver nanoparticles (mg/ml) for some selected pathogenic fungal and bacterial strains.

Name of test NPs	MIC (mg/ml) values					
	Pathogenic fungal strains			Pathogenic bacterial strains		
	*Penicillium Chrysogenum*	*Aspergillus niger*	*Pythium ultimum*	*Staphylococcus aureus*	*Salmonella gallinarum*	*Escherichia coli*
NP MA13^a^	1.0	1.3	0.9	0.8	0.7	0.8
NP MA6^b^	0.6	1.1	1.0	0.6	0.8	0.4

a NP MA13 = AgNPs biosynthesized from *Verpa bohemica.*

b NP MA6= AgNPs mycosynthesized using *Geopora sumneriana* mushroom extract.

## 4 Discussion

Recently, biosynthesized silver nanoparticles (AgNPs) have gained considerable interest owing to their strong antimicrobial activities. They are regarded as safe, eco-friendly, and potential alternatives to conventional antibiotics [32]. In the present study, AgNPs were effectively synthesized using a green approach employing *Verpa bohemica* and *Geopora sumneriana*, and the antibacterial and antifungal activities of mycosynthesized AgNPs and aqueous mushroom extracts were investigated against pathogenic bacterial and fungal strains. Recent studies (Table 5) have demonstrated the potential of macrofungi-mediated green synthesis of silver nanoparticles for diverse biological applications. For instance Ref. [52], reported the synthesis of AgNPs using *Hebeloma excedens*, emphasizing their antidiabetic activity, lipid peroxidation inhibition, and bioaccessibility, thereby highlighting the role of mushroom-derived metabolites in functional nanomaterials. Similarly [53], synthesized AgNPs using *Agrocybe cylindracea* and demonstrated their protective effects against antibiotic-induced lipid and DNA damage. Studies by Refs. [54,55] further explored AgNPs synthesized from *Infundibulicybe gibba* and *Trametes multicolor*, respectively, reporting strong antioxidant, antibacterial, and thermal stability properties, underscoring the importance of fungal biomolecules in nanoparticle bioactivity. Additionally [56], highlighted changes in bioactive components and enhanced antidiabetic potential in AgNPs synthesized from *Melanoleuca grammopodia* (Table 5).

**Table 5:** Comparison of present study with relevant previous reports on fungal-mediated biosynthesis of AgNPs.

Source	Size & morphology	Characterization methods	Reported bioactivities	Relevance	Reference
*Hebeloma excedens* (Basidiomycete)	∼20–60 nm, spherical	UV–Vis, FTIR, TEM, XRD	Antidiabetic, antioxidant	Mushroom metabolites enhanced bioaccessibility and functional activity of AgNPs	[52]
*Agrocybe cylindracea* (Basidiomycete)	∼30–70 nm, spherical	UV–Vis, FTIR, SEM, XRD	Protective against lipid & DNA damage	AgNPs reduced antibiotic-induced oxidative damage	[53]
*Infundibulicybe gibba* (Basidiomycete)	∼25–80 nm	UV–Vis, FTIR, TEM	Antioxidant, antibacterial	Strong role of fungal biomolecules in AgNP bioactivity	[54]
*Trametes multicolor* (Basidiomycete)	∼40–90 nm	UV–Vis, FTIR, SEM, XRD	Antibacterial, thermal stability	Enhanced stability due to protein-mediated capping	[55]
*Melanoleuca grammopodia* (Basidiomycete)	∼20–50 nm	UV–Vis, FTIR, TEM	Antidiabetic	Alteration of bioactive compounds after nanoparticle formation	[56]
Desert truffle mushrooms (Ascomycete)	∼30–100 nm	UV–Vis, SEM, XRD	Antifungal	Strong inhibition of *Pythium* sp., *A. niger*, *A. flavus*	[57]
*Ganoderma lucidum* (Basidiomycete)	∼50 nm, spherical, crystalline	UV–Vis, FTIR, TEM, XRD	Antibacterial, antioxidant	Stable, crystalline AgNPs with broad bioactivity	[45]
*Verpa bohemica* (Ascomycete)	30–50 nm, spherical/hexagonal	UV–Vis, FTIR, SEM, XRD	Antibacterial, antifungal	First report using this species; strong activity against pathogenic bacteria and rot-causing fungi	present study
*Geopora sumneriana* (Ascomycete)	30–50 nm, spherical/hexagonal	UV–Vis, FTIR, SEM, XRD	Antibacterial, antifungal	Novel Ascomycetous source; eco-friendly synthesis with potent antimicrobial efficacy	Present study

During the present study, aqueous extracts did not show antibacterial activity against the tested bacteria, whereas AgNPs exhibited strong antimicrobial efficacy. These results are consistent with those reported by Debnath et al. [42], who observed similar antibacterial responses in AgNPs synthesized from mushroom species. The enhanced antimicrobial activity of AgNPs can be attributed to their unique mechanisms of action, which render them effective even at lower concentrations [32,58]. Apart from bacterial pathogens, fungal infections also pose a serious threat, particularly due to the emergence of resistant strains and the limited availability of new antifungal agents [31]. In comparison, the present study uniquely focuses on underexplored Ascomycetous mushrooms, *Geopora sumneriana* and *Verpa bohemica*, and extends beyond antioxidant or metabolic assessments by providing mechanistic insights into the antimycotic and antibacterial activities of biosynthesized AgNPs. Unlike earlier reports primarily centered on biofunctional or therapeutic attributes, this work emphasizes the dual applicability of fungal-mediated AgNPs in controlling phytopathogenic fungi and pathogenic bacteria, thereby broadening their relevance to agriculture, aquaculture, and horticulture.

The present results are further supported by the findings of Owaid et al. [57], who mycofabricated AgNPs using desert truffle mushrooms and reported potent antifungal activity against *Pythium* sp., *Aspergillus niger*, and *A. flavus*. Similar antifungal efficacy was also documented by Talie et al. [20] and Irshad et al. [59], who observed strong inhibition of pathogenic fungi, including those causing wheat rust disease, by green-synthesized nanomaterials. The biosynthesized AgNPs using *Verpa bohemica* and *Geopora sumneriana* were further evaluated for their minimum inhibitory concentration (MIC) values against different bacterial and fungal pathogens. Comparable investigations were reported by Mohanta et al. [60], Debnath et al. [42], Aygun et al. [61], and Klaus et al. [13], who observed similar MIC ranges and antimicrobial properties in AgNPs synthesized from other mushroom extracts.

During the synthesis process, a characteristic color transformation of the reaction mixture was observed, resulting from surface plasmon resonance (SPR) excitation of the biosynthesized AgNPs [62]. Correspondingly, plasmonic absorption peaks around 420 nm have been reported in AgNPs synthesized from *Agaricus bisporus*, *Helvella leucopus*, and other mushroom species [63,64]. Similar findings were noted by Alfuraydi et al. [65] and Al-Ansari et al. [4], who recorded absorption peaks near 450 nm during AgNP synthesis using other mushroom species. Consistent with our results, Debnath et al. [42] and Owaid et al. [57] reported successful AgNP synthesis using various mushrooms, while Jogaiah et al. [43] and Do-Dat et al. [66] observed spherical and aggregated nanoparticles (20–80 nm) from *Ganoderma applanatum* and *G. lucidum*. Comparable particle morphology and size ranges (20–100 nm) were also described by Nazeruddin et al. [67] and Talie et al. [20]. Likewise, Nguyen et al. [45] reported spherical and crystalline AgNPs (∼50 nm) from *Ganoderma lucidum*, closely matching the current observations.

Further characterization through comparison with the International Centre for Diffraction Data (ICDD) and the Inorganic Crystal Structure Database (ICSD) using PDXL-2 software confirmed the face-centered cubic (fcc) structure and crystalline nature of the biosynthesized AgNPs, consistent with previous reports [24,39]. FTIR analysis confirmed the involvement of fungal biomolecules in the reduction and stabilization of silver nanoparticles synthesized using *Geopora sumneriana* and *Verpa bohemica* extracts. Characteristic bands in the region of 3200–3400 cm^−1^ were attributed to O–H and N–H stretching vibrations of phenolics and proteins, while peaks at 2920–2850 cm^−1^ indicated aliphatic C–H groups. Prominent absorption bands corresponding to amide I (1650–1630 cm^−1^) and amide II (1540–1520 cm^−1^) suggested that proteins play a key role in nanoparticle capping and stabilization. Additional bands between 1250 and 1020 cm^−1^ were assigned to C–O stretching of polysaccharides and phenolic compounds. Notably, shifts in peak positions and variations in intensity compared to crude extracts indicated strong interactions between fungal biomolecules and silver ions, confirming successful biofunctionalization of the AgNPs.

These findings support earlier reports on fungal-mediated nanoparticle synthesis and provide mechanistic evidence for the enhanced stability and antimicrobial efficacy of the biosynthesized AgNPs. Talie et al. [20] and Majeed et al. [68] reported that alcohols, phenolics, proteins, amides, hydroxyl, and carbonyl groups play key roles in capping and reduction processes. Similar observations have been reported by other researchers, who identified amide, carboxylate, hydroxyl, phenolic, and protein groups as stabilizing agents during biological synthesis of AgNPs [11,69,70]. Furthermore, Niraimathi et al. [71], Kharat and Mendhulkar [72], and Poudel et al. [73] demonstrated that carbonyl groups, amines, and proteins possess strong metal-binding capacities, suggesting their vital role as potent capping and reducing agents that stabilize synthesized nanomaterials. Therefore, the current study highlights that *Verpa bohemica* and *Geopora sumneriana* extracts can serve as efficient biological agents for the eco-friendly synthesis of AgNPs. The nanoparticles exhibited excellent antimicrobial and antifungal activities, indicating their potential as novel, cost-effective nanofungicides and nanobactericides.

To elucidate the bioactive components responsible for the reduction, capping, and enhanced antimicrobial activity of biosynthesized silver nanoparticles, detailed biochemical and analytical profiling of fungal extracts can be incorporated [[18], [19], [20]]. Preliminary phytochemical screening may be performed to qualitatively and quantitatively assess phenolics, flavonoids, terpenoids, alkaloids, proteins, polysaccharides, and reducing sugars, which are known to play crucial roles in nanoparticle biosynthesis and stabilization [[21], [22], [23], [24]]. Advanced analytical techniques such as gas chromatography–mass spectrometry (GC–MS) and liquid chromatography–mass spectrometry (LC–MS/MS) can be employed to identify low-molecular-weight secondary metabolites, while high-performance liquid chromatography (HPLC) enables separation and quantification of individual bioactive compounds [51,57,62]. In addition, protein profiling using SDS–PAGE and amino acid analysis can help determine the nature of fungal proteins involved in nanoparticle capping and long-term stability [38,63]. Incorporation of these analyses would provide deeper mechanistic insights into the role of fungal metabolites in nanoparticle formation and bioactivity, thereby strengthening the correlation between fungal chemistry and the observed antimicrobial efficacy of mycosynthesized silver nanoparticles [28,29].

However, several limitations must be acknowledged. First, only a limited number of bacterial and fungal pathogens were tested, restricting a comprehensive understanding of the broad-spectrum efficacy of the synthesized AgNPs. Second, the study was entirely in vitro; therefore, the in vivo safety, toxicity, and stability of the nanoparticles remain untested. Future research should focus on evaluating a wider range of pathogenic and multidrug-resistant microorganisms, developing nanocomposite formulations with other biogenic nanoparticles or natural antimicrobials to enhance spectrum and stability, and investigating environmental impact and biodegradability to ensure the sustainable application of nanomaterials in agro-ecosystems.

## 5 Conclusions

The present study successfully established a simple, cost-effective, and environmentally benign biosynthetic route for the fabrication of silver nanoparticles (AgNPs) using, for the first time, extracts of two novel Ascomycetous mushrooms as efficient reducing and capping agents. Comprehensive characterization using multiple analytical techniques confirmed the successful formation of predominantly spherical to hexagonal, crystalline AgNPs with an average particle size in the range of 30–50 nm, indicating good stability and uniformity. The biofunctionalized AgNPs exhibited pronounced antimycotic and antibacterial activities against rot-causing phytopathogenic fungi as well as clinically relevant pathogenic bacterial strains responsible for severe infectious diseases. The enhanced antimicrobial efficacy can be attributed to the synergistic interaction between fungal-derived bioactive compounds and silver nanoparticles, leading to membrane disruption, oxidative stress, and metabolic interference in microbial cells. Collectively, these findings suggest that AgNPs biosynthesized using Ascomycetous mushroom extracts hold strong potential for development as next-generation nanofungicides and nanobactericides. Such biogenic nanomaterials could offer an effective and sustainable strategy to combat multidrug-resistant microorganisms in agriculture, aquaculture, horticulture, and allied biomedical applications.

## CRediT authorship contribution statement

**Mehrajud Din Talie:** Methodology, Data curation, Conceptualization. **Nusrat Ahmad:** Software, Methodology, Formal analysis, Conceptualization. **Mansoor Ahmad Malik:** Writing – review & editing, Writing – original draft, Investigation. **Abdul Hamid Wani:** Visualization, Validation. **Mohd Yaqub Bhat:** Validation, Supervision.

## Ethical statement

All applicable institutional, national, and international rules and laws were followed in the collecting and processing of fungal materials for this study. This attests to compliance with the relevant legal and ethical requirements for fungal research.

## Funding

The authors received no financial assistance during the study period.

## Declaration of competing interest

The authors declare that they have no known competing financial interests or personal relationships that could have appeared to influence the work reported in this paper.

## Data Availability

The data used in this manuscript is available in the manuscript.
